# Models of kleptoparasitism on networks: the effect of population structure on food stealing behaviour

**DOI:** 10.1007/s00285-017-1177-7

**Published:** 2017-09-18

**Authors:** Christoforos Hadjichrysanthou, Mark Broom, Jan Rychtář

**Affiliations:** 1 Department of Infectious Disease Epidemiology, School of Public Health, Imperial College London, St Mary’s Campus, Norfolk Place, London, W2 1PG UK; 2 Department of Mathematics, City, University of London, Northampton Square, London, EC1V 0HB UK; 3 Department of Mathematics and Statistics, The University of North Carolina at Greensboro, Greensboro, NC 27402 USA

**Keywords:** Kleptoparasitism, Food stealing, Structured populations, Networks, Individual-based models, Pairwise models, 05C57, 91A43, 92B05, 91A40, 91B72, 92D50

## Abstract

The behaviour of populations consisting of animals that interact with each other for their survival and reproduction is usually investigated assuming homogeneity amongst the animals. However, real populations are non-homogeneous. We focus on an established model of kleptoparasitism and investigate whether and how much population heterogeneities can affect the behaviour of kleptoparasitic populations. We consider a situation where animals can either discover food items by themselves or attempt to steal the food already discovered by other animals through aggressive interactions. Representing the likely interactions between animals by a network, we develop pairwise and individual-based models to describe heterogeneities in both the population structure and other individual characteristics, including searching and fighting abilities. For each of the models developed we derive analytic solutions at the steady state. The high accuracy of the solutions is shown in various examples of populations with different degrees of heterogeneity. We observe that highly heterogeneous structures can significantly affect the food intake rate and therefore the fitness of animals. In particular, the more highly connected animals engage in more conflicts, and have a reduced food consumption rate compared to poorly connected animals. Further, for equivalent average level of connectedness, the average consumption rate of a population with heterogeneous structure can be higher.

## Introduction

In many biological situations animals attempt to steal food already discovered by others for their survival. This is a very common form of feeding, usually referred to as *kleptoparasitism*. Different forms of kleptoparasitic behaviour are observed in many species in the animal kingdom, for example species of spiders (e.g., Coyle et al. 1991), birds (e.g., Brockmann and Barnard 1979), snails (e.g., Iyengar 2002), lizards (e.g., Cooper and Pérez-Mellado 2003), fish (e.g., Hamilton and Dill 2003), primates (e.g., Janson 1985), carnivores (e.g., Carbone et al. 2005) and insects (e.g., Erlandsson 1988). This behaviour of animals has been well documented in a review paper (Iyengar 2008).

There are a number of theoretical models focused on the kleptoparasitic behaviour of animals using different mathematical methods, in particular evolutionary game theory (e.g., Barnard and Sibly 1981; Broom and Ruxton 1998). The model of Broom and Ruxton (1998) is a model of kleptoparasitism which employs game theory to consider the ecological conditions under which attacking to steal the food from other animals when the opportunity arises is the best strategy that foraging animals should adopt in order to maximise their food intake rate and consequently their fitness. Food in this model comes as single indivisible items, which must be consumed completely by an animal. Thus, food can never be shared and challenging animals attempt to steal the whole item from the owner, or not.

A series of publications has appeared developing the original model of Broom and Ruxton (1998) in a number of ways (e.g., Broom and Ruxton 2003; Broom and Rychtář 2007; Luther et al. 2007; Yates and Broom 2007; Broom et al. 2008; Broom and Rychtář 2009, 2011; Hadjichrysanthou and Broom 2012). Crowe et al. (2009) provides a brief review on the main theoretical work on kleproparasitism prior to the investigation of a stochastic model of kleptoparasitism in finite populations. A comparison between some main models of kleptoparasitism is discussed in Vahl (2006) and an alternative model is presented. There is also a series of related mechanistic, but not game-theoretic, models which investigate interference competition, where foraging animals engage in aggressive interactions in order, for example, to defend their territory, resulting in negative effects on their foraging efficiency (e.g., Beddington 1975; Ruxton et al. 1992; van der Meer and Ens 1997; Vahl 2006; Smallegange and van der Meer 2009; van der Meer and Smallegange 2009).

The game-theoretical model of Broom and Ruxton (1998) and the subsequent work assumed that the population of foraging animals is infinitely large, homogeneous and well-mixed, where every animal has the same foraging and fighting abilities and is equally likely to meet and interact with any other animal. However, in natural situations, animals differ in these abilities and usually forage in small groups forming complex relationships and social structure (e.g., Krause et al. 2007; Croft et al. 2008). A number of stochastic models have been developed to consider the dynamics of kleptoparasitic populations of finite size (see Yates and Broom 2007; Crowe et al. 2009). However, the effect of the population structure and other heterogeneities on the behaviour of such populations remains a research question.

In this paper, we explore the role of the underlying connectivity and other heterogeneities between animals in the dynamics of kleptoparasitic populations. Interactions between animals are represented by a static network; each animal is assumed to occupy a node, the links between nodes represent interactive relationships. We first revisit the original model of Broom and Ruxton (1998), which provides a baseline approximation to the key features of the system that we will consider, in particular the handling ratio, the proportion of animals handling food items at any one time. We then extend the model to networks. We develop and analyse an individual-based model which incorporates an arbitrarily complex population structure and heterogeneities in the model parameters and obtain a more precise estimate of these features, albeit with the downside that a large number of parameters and distinct estimates are required. To reduce the number of parameters and equations we also describe the system by a pairwise approximation model. We consider the effect of the population structure and compare the numerical solution of the two models and analytical solutions derived with the results of stochastic simulations on theoretical and empirical networks.

## Models of kleptoparasitism in homogeneous well-mixed populations

### The model of Broom and Ruxton (1998)

In the basic model of Broom and Ruxton (1998) each of the animals in a population of foragers either searches for food, has already acquired and is handling a food item prior to its consumption, or fights with another animal over a food item. Let us denote by *P* the population density, by *S* the density of searchers and by *H* the density of handlers. When a foraging animal encounters an animal in the handling state it attacks it to steal the prey. There is a constant density of food items *f* available and searchers cover an area $\nu _{f}$ per unit of time whilst searching for food, so animals find food at rate $\nu _{f}f$. If a handler animal is not attacked, it consumes its food item in a time drawn randomly from an exponential distribution with mean $t_h$. Attacked animals always defend their food and a fight takes place. Searchers encounter handlers and engage in a fight at rate $\nu _{h}H$. A fight lasts for a time drawn randomly from an exponential distribution with mean $t_a/2$. In the model of Broom and Ruxton (1998) it was assumed that animals involved in an aggressive interaction are equally likely to win the fight and obtain the food. Here, we allow different competitive abilities between the attacker and the attacked animals, i.e. the probability of the attacking animal winning and obtaining the food, $\alpha$, varies between 0 and 1, as happens in natural situations. This was an extension to the original model introduced by Broom et al. (2004). We denote by *A* and *R* the density of attacking searchers and defenders engaged in a fight, respectively. The loser of the fight returns to the searching state while the winner starts handling the food item. The model notation is summarised in Table 1.

**Table 1:** Notation of the basic game-theoretical model of kleptoparasitism

Population’s densities	Meaning
*P*	Density of the population
*S*, *H*, *A*, *R*	Density of searchers, handlers, attackers and defenders

Model parameters	Meaning
$\nu _{f}f$	Rate at which foragers find undiscovered food
$\nu _{h}H$	Rate at which foragers encounter handlers
$t_{h}$	Expected time for a handler to consume a food item if it is not attacked
$t_{a}/2$	Expected duration of a fight
$\alpha$	The probability that the attacker wins the fight

The system of equations constructed to describe the dynamics of the four subpopulations (see Ruxton and Moody 1997; Broom and Ruxton 1998; Broom et al. 2004) is the following:

1 $$\begin{aligned} \frac{{ dS}}{{ dt}}= & {} \frac{1}{t_h}H+\frac{2}{t_a}(1-\alpha )A+\frac{2}{t_a}\alpha R-\nu _{f}{} { fS}-\nu _{h}{} { SH}, \end{aligned}$$

2 $$\begin{aligned} \frac{{ dH}}{{ dt}}= & {} \nu _{f}{} { fS}+\frac{2}{t_a}\alpha A +\frac{2}{t_{a}}(1-\alpha )R-\frac{1}{t_h}H-\nu _{h}{} { SH}, \end{aligned}$$

3 $$\begin{aligned} \frac{{ dA}}{{ dt}}= & {} \nu _{h}{} { SH}-\frac{2}{t_a}A, \end{aligned}$$

4 $$\begin{aligned} \frac{{ dR}}{{ dt}}= & {} \nu _{h}{} { SH}-\frac{2}{t_a}R. \end{aligned}$$

### The searchers-handlers relationship at the steady state and the handling ratio

At the steady state of the system (1)–(4) the searching population is proportional to the handling population:

5 $$\begin{aligned} S = \frac{H}{t_hv_ff}. \end{aligned}$$

One of the most important quantities is the food intake rate $\gamma$, which is a natural measure of the payoff for an animal. This is given by

6 $$\begin{aligned} \gamma =\frac{H}{t_hP}, \end{aligned}$$

where the *handling ratio* $\frac{H}{P}$, the proportion of handlers at the steady state, is given by

7 $$\begin{aligned} \frac{H}{P}=\frac{-(t_h\nu _{f}f+1)+\sqrt{(t_h\nu _{f}f+1)^2+4t_ht_a\nu _{f}f\nu _{h}P}}{2t_a\nu _{h}P}. \end{aligned}$$

Equation (7) is the baseline of the handling ratio that will be compared with the handling ratios in subsequent models.

## Models of kleptoparasitism on networks

### An individual-based model

We extend the model (1)–(4) at the individual-level to allow heterogeneity in the parameters and the underlying connectivity between animals. We assume that the population can be represented by a network of *N* nodes. Each node is occupied by a single animal. A link between two nodes represents a connection between the two animals occupying the nodes. The network can be described by the adjacency matrix $A_M=[a_{ij}]$, where $a_{ij} = 1$ if node *i* is connected to node *j* and $a_{ij} = 0$ otherwise, $i, j \in \{1, 2,\ldots , N\}$. All the networks that we consider in this study are static networks. It is also natural to assume that the networks are undirected, i.e. an animal that can attack another animal can also be attacked by the same animal.

One of the important quantities that characterises an individual in the network is its degree, which measures the number of its nearest neighbours. In an undirected network, the degree of node *i*, $d_i$, is given by:

8 $$\begin{aligned} d_i = \sum _j a_{ij} = \sum _j a_{ji}. \end{aligned}$$

An animal at node *i* is either in the searching state with probability ${\langle S_i\rangle }$, the handling state with probability ${\langle H_i\rangle }$, or it is fighting with a connected animal in some node *j*. Animal *i* searches for food at speed ${v_f}_i$ and discovers a food item at rate ${v_f}_if_i$, where $f_i$ is the food availability for the animal in node *i*. A searcher $S_i$ also searches for handlers at rate $v_{h,i}$. If a searcher encounters a handler, they engage in a fight over the food. Let ${\langle A_{i}R_{j}\rangle }$ denote the probability that animals in nodes *i* and *j* are engaged in a fight following the attack of $S_i$ on $H_j$. Clearly, $\sum _{j}{\langle A_{i}R_{j}\rangle }$ is equal to the overall probability of an animal in node *i* being an attacker, $\langle A_{i}\rangle$, and $\sum _{i}{\langle A_{i}R_{j}\rangle }$ is equal to the overall probability of an animal in node *j* being a defender, $\langle R_{j}\rangle$. The expected fight duration between a searcher *i* and a handler *j* is $t_{a,ij}/2$. The fight time $t_{a,ij}/2$ might differ from $t_{a,ji}/2$; for example the length of the contest might depend upon the persistence of the attacking animal, which can be different in the two cases. At the end of the fight between animals in nodes *i* and *j*, the attacking searcher wins with probability $\alpha _{ij}$ and takes the food. A handler consumes the food at rate $t_{h,i}^{-1}$, thus taking an expected time of $t_{h,i}$ to consume the item, and then returns back to the searching state.

The dynamics of the system can be described by the following model:

9 $$\begin{aligned} \frac{d \langle S_i \rangle }{{ dt}}= & {} -v_ff_i\langle S_i \rangle + t_{h,i}^{-1} \langle H_i \rangle - v_{h,i}\langle S_i \rangle \sum _j a_{ij}\langle H_j\rangle \nonumber \\&+\,2\sum _j (1-\alpha _{ij})t_{a,ij}^{-1}\langle A_{i}R_{j} \rangle + 2\sum _j \alpha _{ji}t_{a,ji}^{-1}\langle A_{j}R_{i}\rangle , \end{aligned}$$

10 $$\begin{aligned} \frac{d \langle H_i \rangle }{{ dt}}= & {} v_ff_i\langle S_i \rangle - t_{h,i}^{-1} \langle H_i \rangle - \langle H_i \rangle \sum _j a_{ji}v_{h,j}\langle S_j \rangle \nonumber \\&+\,2\sum _j \alpha _{ij}t_{a,ij}^{-1} \langle A_{i}R_{j} \rangle + 2\sum _j (1-\alpha _{ji})t_{a,ji}^{-1}\langle A_{j}R_{i}\rangle , \end{aligned}$$

11 $$\begin{aligned} \frac{d \langle A_{i}R_{j} \rangle }{{ dt}}= & {} a_{ij}v_{h,i}\langle S_i \rangle \langle H_j \rangle - 2t_{a,ij}^{-1} \langle A_{i}R_{j} \rangle . \end{aligned}$$

It should be noted that $v_{h,i}$ and $a_{ij}$ always appear as a product, i.e. as $v_{h,i}a_{ij}$, $\forall i,j\in \{1,\ldots , N\}$, with the product representing the rate at which a searcher animal *i* encounters a handler *j* during the time it invests in searching for handlers. Hence, we could simply assume that the adjacency matrix is a weighted matrix $A_M'$ whose element $a'_{i,j}$ is equal to $v_{h,i}a_{ij}$, $\forall i,j\in \{1,\ldots , N\}$. However, to be consistent with the notation of previous models developed we separate the two terms. In addition, as ${v_f}_i$ and $f_i$ always appear as a product, we have replaced ${v_f}_if_i$ by $v_ff_i$.

The system (9)–(11) consists of $N(2+d)$ equations, where *d* is the average degree of a node. This system assumes statistical independence between animals that are not fighting each other. This assumption can be relaxed by extending the system further to include the dynamics of pairs of animals at the searching, handling and fighting state (see for example Sharkey 2008 for relevant epidemic models on networks). However, such complex extensions are more computationally expensive and as the model (9)–(11) is very accurate (see Sect. 4), we focus on this to study the effect of the population structure in kleptoparasitic populations as described by Broom and Ruxton (1998).

#### Steady states of the individual-based model and the handling ratio

In Luther and Broom (2004) the authors proved rapid convergence to a unique solution for the considerably simpler original models of Ruxton and Moody (1997) and Broom and Ruxton (1998). Despite the complexity of the system (9)–(11) we believe that this has a unique steady-state solution too. Although the complexity of the system has meant that we are unable to prove this, the output of stochastic simulations supports this statement.

At the steady state:

12 $$\begin{aligned} 0&= -v_ff_i\langle S_i \rangle + t_{h,i}^{-1} \langle H_i \rangle - v_{h,i}\langle S_i \rangle \sum _j a_{ij}\langle H_j\rangle \nonumber \\&\quad + 2\sum _j (1-\alpha _{ij})t_{a,ij}^{-1}\langle A_{i}R_{j} \rangle + 2\sum _j \alpha _{ji}t_{a,ji}^{-1}\langle A_{j}R_{i} \rangle , \end{aligned}$$

13 $$\begin{aligned} 0&= v_ff_i\langle S_i \rangle - t_{h,i}^{-1} \langle H_i \rangle - \langle H_i \rangle \sum _j a_{ji}v_{h,j}\langle S_j \rangle \nonumber \\&\quad + 2\sum _j \alpha _{ij}t_{a,ij}^{-1} \langle A_{i}R_{j} \rangle + 2\sum _j (1-\alpha _{ji})t_{a,ji}^{-1}\langle A_{j}R_{i} \rangle , \end{aligned}$$

14 $$\begin{aligned} 0&= a_{ij}v_{h,i}\langle S_i \rangle \langle H_j \rangle - 2t_{a,ij}^{-1} \langle A_{i}R_{j} \rangle . \end{aligned}$$

From (14) it follows that at the steady state

15 $$\begin{aligned} \langle A_{i}R_{j} \rangle = \frac{t_{a,ij}}{2} a_{ij}v_{h,i}\langle S_i \rangle \langle H_j \rangle . \end{aligned}$$

Substituting Eq. (15) into (13) we obtain:

16 $$\begin{aligned} \langle H_i \rangle = t_{h,i}v_ff_i\langle S_i \rangle \frac{v_{h,i}\sum _j \alpha _{ij}a_{ij}\langle H_j\rangle + v_ff_i}{t_{h,i}v_ff_i\sum _j \alpha _{ji}a_{ji} v_{h,j}\langle S_j\rangle + v_ff_i}. \end{aligned}$$

We will be considering an approximation solution of the system (12)–(14) by assuming that

17 $$\begin{aligned} \langle S_i\rangle = \frac{\langle H_i\rangle }{t_{h,i}v_ff_i},\quad \forall i\in \{1,\ldots , N\}. \end{aligned}$$

Equation (17) satisfies Eq. (16) in homogeneous well-mixed populations (see also Eq. (5)). This relationship between searchers and handlers has been shown to be valid in such populations also in previous work (Broom and Ruxton 1998; Broom et al. 2004), as well as in subsequent network-based models considered in this paper (see Sect. 3.2).

Since an animal is always in one of the three states, searching, handling or fighting, we have that

18 $$\begin{aligned} \langle S_i \rangle + \langle H_i\rangle + \sum _j \langle A_{i}R_{j}\rangle + \sum _j \langle A_{j}R_{i}\rangle = 1. \end{aligned}$$

Substituting (15) and (17) into (18) we get

19 $$\begin{aligned} \langle H_i\rangle \left[ \frac{1}{t_{h,i}v_ff_i}+1 + \frac{1}{2}\sum _j \left( \frac{t_{a,ij}a_{ij}v_{h,i}}{t_{h,i}v_ff_i} + \frac{t_{a,ji}a_{ji}v_{h,j}}{t_{h,j}v_ff_j}\right) \langle H_j\rangle \right] =1, \end{aligned}$$

where we assumed that $v_ff_i>0$ for all $i\in \{1,\ldots , N\}$.

(19) is a system of *N* simultaneous equations with *N* unknowns, the individual handling ratios, which are the probabilities of each animal handling a food item at any time point at the steady state.

A potential limitation of the solution of Eq. (19), which is based on the assumption (17), is that this is independent of the probability of an attacking searcher *i* winning the fight with handler *j*, $\alpha _{ij}$, $\forall i,j\in \{1,\ldots , N\}$. However, as discussed in Sect. 4, the value of the probability $\alpha _{ij}$, $\forall i,j\in \{1,\ldots , N\}$, does not affect the system in homogeneous populations placed on complete or regular networks and its effect on the average handling ratio in populations placed on heterogeneous networks, if any, is negligible.

The mean handling ratio of the system (9)–(11) is given by

20 $$\begin{aligned} \overline{H}=\frac{1}{N} \sum _{i=1}^{N}\langle H_i\rangle . \end{aligned}$$

#### A special case: a homogeneous population on a regular undirected network

In the case of a homogeneous population placed on a regular undirected network, Eq. (19) is solved by

21 $$\begin{aligned} \langle H_i\rangle = \langle H\rangle ,\quad \forall i\in \{1,\ldots , N\}. \end{aligned}$$

Substituting (21) into (19) yields

22 $$\begin{aligned} t_{a}\frac{v_h}{t_hv_ff}d \langle H\rangle ^2 + \left( \frac{1}{t_hv_ff}+1 \right) \langle H\rangle -1=0, \end{aligned}$$

where *d* is the degree of each of the nodes. The biologically relevant solution of the above equation is

23 $$\begin{aligned} \langle H\rangle =\frac{-(t_h\nu _{f}f+1)+\sqrt{(t_h\nu _{f}f+1)^2+4t_ht_a\nu _{f}f\nu _{h}d}}{2t_a\nu _{h}d}. \end{aligned}$$

We observe that for $d=P$, (23) is identical to Eq. (7). In other words, setting $\nu _{h}=d$ in the case of a homogeneous well-mixed population (system (1)–(4)) with $P=1$, yields equivalent results to those obtained in the case of a structured population represented by a regular network with degree *d*. Hence, decreasing the number of neighbouring animals in a homogeneous population placed on a regular network has the same effect as decreasing the rate at which foragers encounter handlers in a homogeneous well-mixed population, i.e. as decreasing $\nu _{h}$.

### A pairwise model

Although the individual-based model developed in the previous section can describe more realistic systems, it is characterised by a large number of variables and parameters. In this section, to reduce complexity, we consider an alternative model for the description of the dynamics of a structured kleptoparasitic population. This is a pairwise model developed using the pair approximation method (Matsuda et al. 1992; van Baalen and Rand 1998; Keeling 1999; Eames and Keeling 2002; House and Keeling 2011). The method assumes regularity of the network and identical parameters for every individual.

Assume that animals of a finite homogeneous population occupy the nodes of a regular network of degree *d*, i.e. every animal has exactly *d* neighbours. Let [*X*] be the number of animals in state *X*, [*XY*] the number of $X-Y$ pairs between an animal in state *X* and an animal in state *Y*, and [*XYZ*] the number of triples of type $X-Y-Z$. *X*, *Y* and *Z* represent any of the foraging states; the searching state *S*, the handling state *H*, the attacking state *A* and the resisting state *R*. Two connected animals in the fighting state might fight each other or they might fight with some other animal. We distinguish these different types of pairs of animals by denoting by $[AR_J]$ the number of pairs of animals which are fighting each other, and by [*AR*], [*AA*] and [*RR*] the number of pairs of animals which are involved in a fight, either by attacking or resisting, but which are not fighting each other (so that both, one or neither could be an *A*). $X-X$ pairs are counted twice (once in each direction, and thus [*XX*] is always even), whereas $X-Y$ pairs are counted once in each direction, and $[XY]=[YX]$.

Following the rules of the game as described in Sect. 2.1, the dynamics of the singles and pairs can be described by the following system of differential equations: [1]

24 $$\begin{aligned} \frac{d[S]}{{ dt}}&= \frac{1}{t_h}[H]+\frac{2}{t_a}(1-\alpha )[A]+\frac{2}{t_a}\alpha [R]-\nu _{f}f[S]-\nu _{h}[{ SH}], \end{aligned}$$

25 $$\begin{aligned} \frac{d[H]}{{ dt}}&= \nu _{f}f[S]+\frac{2}{t_a}\alpha [A]+\frac{2}{t_a}(1-\alpha )[R]-\frac{1}{t_h}[H]-\nu _{h}[{ SH}], \end{aligned}$$

26 $$\begin{aligned} \frac{d[A]}{{ dt}}&= \nu _{h}[{ SH}]-\frac{2}{t_a}[A], \end{aligned}$$

27 $$\begin{aligned} \frac{d[R]}{{ dt}}&= \nu _{h}[{ SH}]-\frac{2}{t_a}[R], \end{aligned}$$

28 $$\begin{aligned} \frac{d[{ SS}]}{{ dt}}&= \frac{2}{t_h}[{ SH}]+\frac{4}{t_a}(1-\alpha )[{ SA}]+\frac{4}{t_a}\alpha [{ SR}]-2\nu _{f}f[{ SS}]-2\nu _{h}[{ SSH}], \end{aligned}$$

29 $$\begin{aligned} \frac{d[{ HH}]}{{ dt}}&=2\nu _{f}f[{ SH}]+\frac{4}{t_a}\alpha [{ HA}]+\frac{4}{t_a}(1-\alpha )[{ HR}]-\frac{2}{t_h}[{ HH}]-2\nu _{h}[{ HHS}], \end{aligned}$$

30 $$\begin{aligned} \frac{d[{ AA}]}{{ dt}}&= 2\nu _{h}[{ ASH}]-\frac{4}{t_a}[{ AA}], \end{aligned}$$

31 $$\begin{aligned} \frac{d[{ AR}]}{{ dt}}&= \nu _{h}[{ HSR}]+\nu _{h}[{ AHS}]-\frac{4}{t_a}[{ AR}], \end{aligned}$$

32 $$\begin{aligned} \frac{d[{ RR}]}{{ dt}}&= 2\nu _{h}[{ RHS}]-\frac{4}{t_a}[{ RR}], \end{aligned}$$

33 $$\begin{aligned} \frac{d[{ AR}_J]}{{ dt}}&= 2\nu _{h}[{ SH}]-\frac{2}{t_a}[{ AR}_J], \end{aligned}$$

34 $$\begin{aligned} \frac{d[{ SH}]}{{ dt}}&= \nu _{f}f[{ SS}]+\frac{1}{t_h}[{ HH}]\nonumber \\&\quad +\frac{2}{t_a}\left( \alpha [{ SA}]+(1-\alpha )[{ SR}]+(1-\alpha )[{ AH}]+\alpha [{ RH}]+\frac{1}{2}[{ AR}_J]\right) \nonumber \\&\quad -\left( \nu _{f}f+\frac{1}{t_h}\right) [{ SH}]-\nu _{h}\left( [{ SH}]+[{ SHS}]+[{ HSH}]\right) , \end{aligned}$$

35 $$\begin{aligned} \frac{d[{ SA}]}{{ dt}}&= \frac{1}{t_h}[{ HA}]+\frac{2}{t_a}(1-\alpha )[{ AA}]+\frac{2}{t_a}\alpha [{ RA}]+\nu _{h}[{ SSH}]\nonumber \\&\quad -\left( \nu _{f}f+\frac{2}{t_a}\right) [{ SA}]-\nu _{h}[{ ASH}], \end{aligned}$$

36 $$\begin{aligned} \frac{d[{ SR}]}{{ dt}}&= \frac{1}{t_h}[{ HR}]+\frac{2}{t_a}(1-\alpha )[{ AR}]+\frac{2}{t_a}\alpha [{ RR}]+\nu _{h}[{ SHS}]\nonumber \\&\quad -\left( \nu _{f}f+\frac{2}{t_a}\right) [{ SR}]-\nu _{h}[{ HSR}], \end{aligned}$$

37 $$\begin{aligned} \frac{d[{ HA}]}{{ dt}}&= \nu _{f}f[{ SA}]+\frac{2}{t_a}\alpha [{ AA}]+\frac{2}{t_a}(1-\alpha )[{ RA}]+\nu _{h}[{ HSH}]\nonumber \\&\quad -\left( \frac{1}{t_h}+\frac{2}{t_a}\right) [{ HA}]-\nu _{h}[{ SHA}], \end{aligned}$$

38 $$\begin{aligned} \frac{d[{ HR}]}{{ dt}}&= \nu _{f}f[{ SR}]+\frac{2}{t_a}\alpha [{ AR}]+\frac{2}{t_a}(1-\alpha )[{ RR}]+\nu _{h}[{ HHS}]\nonumber \\&\quad -\left( \frac{1}{t_h}+\frac{2}{t_a}\right) [{ HR}]-\nu _{h}[{ SHR}]. \end{aligned}$$

We ‘close’ the system of equations at the level of pairs by approximating the triples by an expression in terms of singles and pairs. The number of the triples $[{ XYZ}]$ can be approximated by the following moment closure approximation (see for example, Keeling 1999; Rand 1999):

39 $$\begin{aligned}{}[{ XYZ}]=\left( \frac{d-1}{d}\right) \frac{[{ XY}][{ YZ}]}{[Y]}, \end{aligned}$$

where *d* is the degree of each node in the network.

Hence, the system (24)–(38), together with the closure approximation (39), is a system of 15 differential equations, which is significantly lower than the number of equations in the individual-based model (9)–(11), which has two equations for every individual and for every link.

Clearly, since for every animal *A* there is one animal *R*,

40 $$\begin{aligned}{}[A]=[R]=[{ AR}_J]. \end{aligned}$$

In addition, at the steady state, at which all the equations in the system (9)–(11) are equal to zero, from Eq. (26) we get

41 $$\begin{aligned}{}[A]=\frac{t_a}{2}\nu _{h}[{ SH}]. \end{aligned}$$

Substituting Eqs. (40) and (41) into (25) we get that at the steady state

42 $$\begin{aligned}{}[H]=t_h\nu _{f}f[S]. \end{aligned}$$

Since

43 $$\begin{aligned}{}[S]+[H]+[A]+[R]=N, \end{aligned}$$

using (40), (41) and (42) we get

44 $$\begin{aligned}{}[S]=\frac{N-t_a\nu _{h}[{ SH}]}{1+t_h\nu _{f}f}. \end{aligned}$$

The steady state solution of the system (24)–(39) is derived in “Appendix A”.

## Numerical examples: effect of network structure on the handling ratio

We compare the solution of the models developed in the previous section with the results of stochastic simulations on a number of theoretical and empirical networks; a random network, a random regular network, a scale-free network and the Guppy network. The stochastic simulation is described in “Appendix B”. The random network we consider is an Erdős–Rényi type network (Erdős and Rényi 1959) generated as described in Lindquist et al. (2011). The random regular network was generated in the same way as the random network with the restriction that every vertex has the same number of connections. The scale-free network is a network that has a power-law (or scale-free) degree distribution. This was generated following the algorithm of preferential attachment (Barabási and Albert 1999; Albert and Barabási 2002). The random, random regular and scale-free networks developed are undirected and unweighted. Unless otherwise stated, the number of nodes of each of these networks that are considered in the current study is 100 and the average degree is 4. The Guppy network is a real network dataset that represents the contacts of a well-known freshwater fish species, the guppy (*Poecilia reticulata*), also known as the millionfish (Croft et al. 2011). This network is complex and highly structured, and exhibits small-world network properties (Watts and Strogatz 1998). It is a weighted network consisting of 99 nodes and 726 undirected links, where each node represents a guppy and each link a social interaction between two guppies. The average connectivity of each node is 14.7 and the clustering coefficient (Watts and Strogatz 1998) of the network is 0.77. To be consistent with the other example networks considered in this work, the Guppy network was converted to an unweighted network. In Croft et al. (2011) there is a detailed consideration of some local and global parameters of the Guppy network, such as the average geodesic (the smallest number of edges by which one node can be reached from the other), and other social characteristics. To illustrate the effect of the population structure in each of the examples, the solution of the models on the complete network (with $N=100$), where every animal is connected to everyone else, is also presented and compared with the other solutions. Clearly, in large homogeneous well-mixed populations all models become equivalent and exact.

### Case 1: identical parameter values among individuals

We assume the same parameter values for every individual and compare the numerical solutions of the individual-based and pairwise models with the results of stochastic simulations. We also evaluate and compare all the approximate steady state solutions for both models, i.e. the solution of (19), of Eq. (23) and of the equation for [*H*] in (60). Since (23) is a solution of the system (19) in the special case of homogeneous populations on regular undirected networks, the solutions of (19) and (23) are equivalent on well-mixed and random regular networks.

**Fig. 1:**
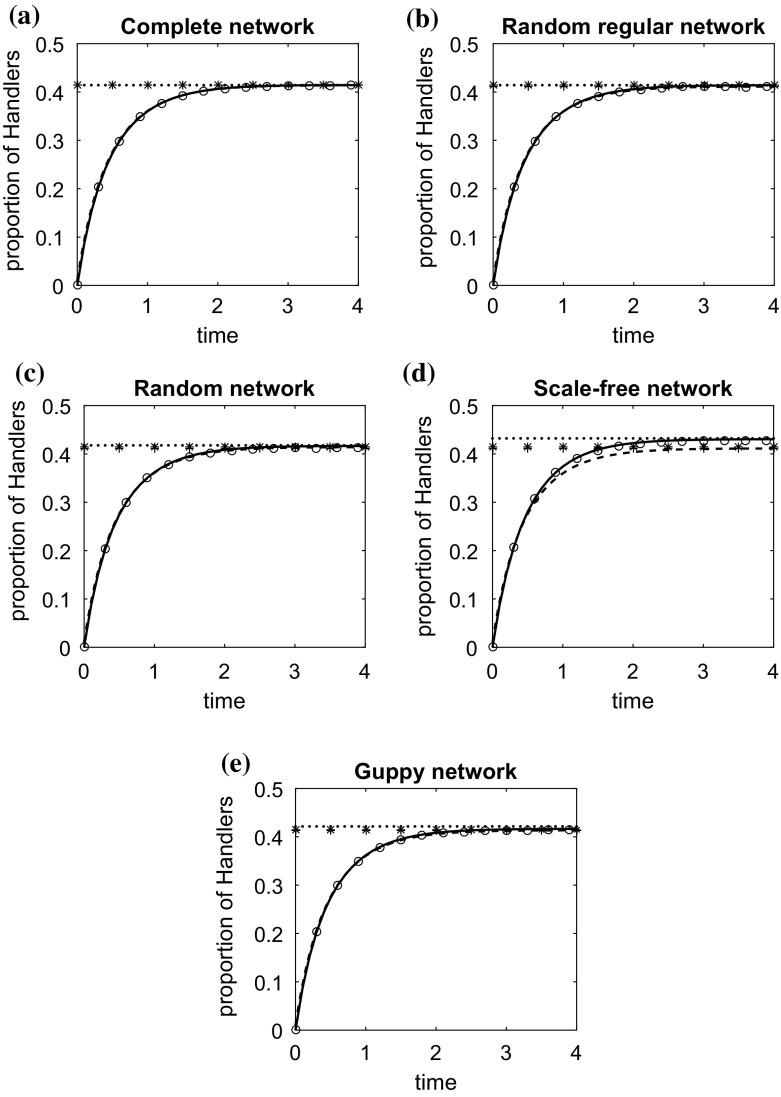
Change over time in the density of handlers on **a** a complete network, **b** a random regular network, **c** a random network, **d** a scale-free network, **e** the Guppy network. The characteristics of these networks are discussed in Sect. 4. The circles represent the average of 10,000 stochastic simulations. Initially, at $\hbox {time}=0$, all animals are at the searching state. The solid line is the solution of the individual-based model (9)–(11) and the dashed line the solution of the pairwise model (24)–(39). The dotted line and the asterisks, ‘*’, are respectively the approximate solutions (19) and (23) of the individual-based model at the steady state. The plus signs, ‘$+$’, is the solution of the pairwise model explicitly given in “Appendix A”. $P=1, t_{a,ij}/2=0.5, \alpha _{ij}=0.5, t_{h,i}=1, v_ff_i=1, v_{h,i}=1/d_i, i,j \in \{1,\ldots , N\}$

**Fig. 2:**
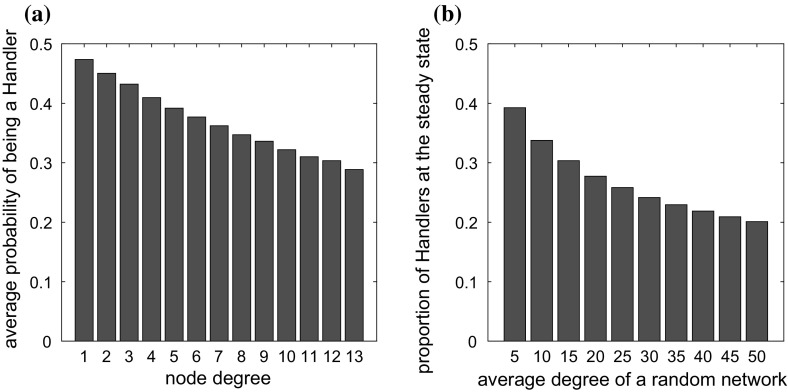
**a** The average probability of an animal being a handler at the steady state as a function of the degree of the node that it occupies on a random network with average degree equal to 6 and $N=100$. **b** The density of handlers at the steady state on random networks of different degree and $N=100$. The results have been derived by solving the individual-based model (9)–(11) at the steady state. $t_{a,ij}/2=0.5, \alpha _{ij}=0.5, t_{h,i}=1, v_ff_i=1, v_{h,i}=0.3, i,j \in \{1,\ldots , N\}$

In Fig. 1, we illustrate the variation in the density of handlers over time on the different networks when $v_{h,i}= v_h/d_{i}$. This example can describe the case where the search for handlers effectively requires a time consuming action such as a ‘visit’ to a neighbour’s site, where $v_h$ is some basic rate at which animals can perform the search. It is observed that the density of handling animals (and similarly animals in other states) is not changed significantly by the change in the population structure, especially when animals are placed on a regular (or at least not a highly-structured) network. This is mainly because the number of connections is the same (or almost the same) for every animal, and therefore every animal has the same chance of engaging in an aggressive interaction.

A more pronounced structural effect is observed when the structure is highly heterogeneous, for example a structure which has the features of a scale-free network (see Fig. 1d). The existence of animals whose degree greatly exceeds the average reduces significantly the overall number of fights taking place over food, resulting in important changes of the population food intake rate. In particular, the increased number of connections of an animal increases its chance to be involved in a fight over food with another animal. This has a negative effect on its food consumption rate (see for example Fig. 2a). On the other hand, lowly connected animals can search, find and consume food with a very small risk of being engaged in an aggressive interaction. Since in networks with a power-law degree distribution the number of highly connected nodes is small, the average handling ratio, and thus the food intake rate of the population, increases compared to that of the respective well-mixed population of the same size, or the infinite homogeneous well-mixed population of the model of Broom and Ruxton (1998).

In regular and random networks, both the individual-based model and the pairwise model agree very well with the results of simulations. In fact the pairwise model seems to perform slightly better than the individual-based model on regular networks; this is reasonable as it is designed for this type of networks, whereas the individual-based model makes some assumptions of statistical independence. In highly heterogeneous structures, the solution of the individual-based model and its approximate solution at the steady state, (19), perform much better than the respective solutions of the pairwise model (see Fig. 1d).

**Fig. 3:**
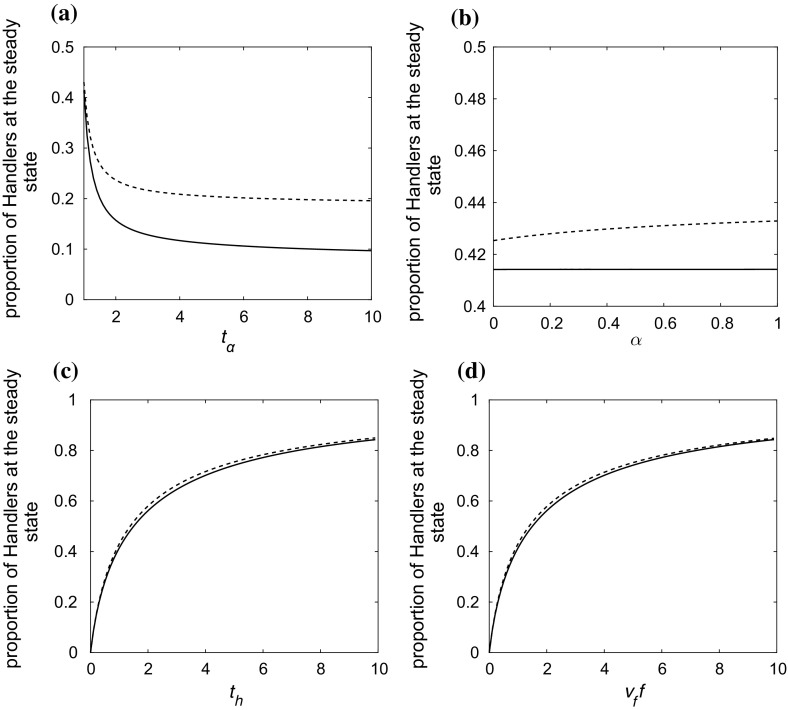
The effect of the change of the parameters **a** $t_{a,ij}$, **b** $\alpha _{ij}$, **c** $t_{h,i}$, **d** $v_ff_i$, on the proportion of handlers at the steady state on a scale-free (dashed line) and a complete (solid line) network of $N=100$. In each case, a single parameter is varied, and all other parameters remain fixed. The results have been derived by solving the individual-based model (9)–(11) at the steady state. In every case, $t_{a,ij}=t_a, \alpha _{ij}=\alpha , t_{h,i}=t_h, v_ff_i=\nu _{f}f, v_{h,i}=1/d_i, i,j \in \{1,\ldots , N\}$. When $t_a, \alpha , t_h$ and $\nu _{f}f$ are fixed, $t_a=1, \alpha =0.5, t_h=1$ and $\nu _{f}f=1$

It should be noted that the rate at which foragers find undiscovered food, $\nu _{f}f$, and the expected time for a handler to consume a food item, $t_{h}$, seem to have the most important influence on the average intake rate of kleptoparasitic populations irrespective of their structure. However, the duration of a fight between two animals, $t_{a}/2$, is the parameter whose increase yields higher differences between the average intake rate in structured populations and that in homogeneous well-mixed populations (see Fig. 3). The choice of the value of the probability of an attacking searcher *i* winning the fight with a defender *j*, $\alpha _{ij}$, is generally insignificant (see for example Fig. 3b). In particular, in homogeneous well-mixed populations, after any contest there is one winner and one loser both connected to the same number of animals and thus the population distribution at the steady state is the same irrespective of $\alpha _{ij}$, $i, j \in \{1, 2,\ldots , N\}$. The solution of the pairwise model at the steady state found in “Appendix A”, which we have not conclusively proved but we believe to be the unique solution, is also independent of the value of $\alpha$. Moreover, even in populations with extreme structural heterogeneity different values of $\alpha$ make little or no difference; although the value of $\alpha$ can have an effect on the handling ratio of each animal individually, the change of the average handling ratio, if any, is negligible. This is verified both by the results of stochastic simulations and the numerical solution of the individual-based model (9)–(11).

**Fig. 4:**
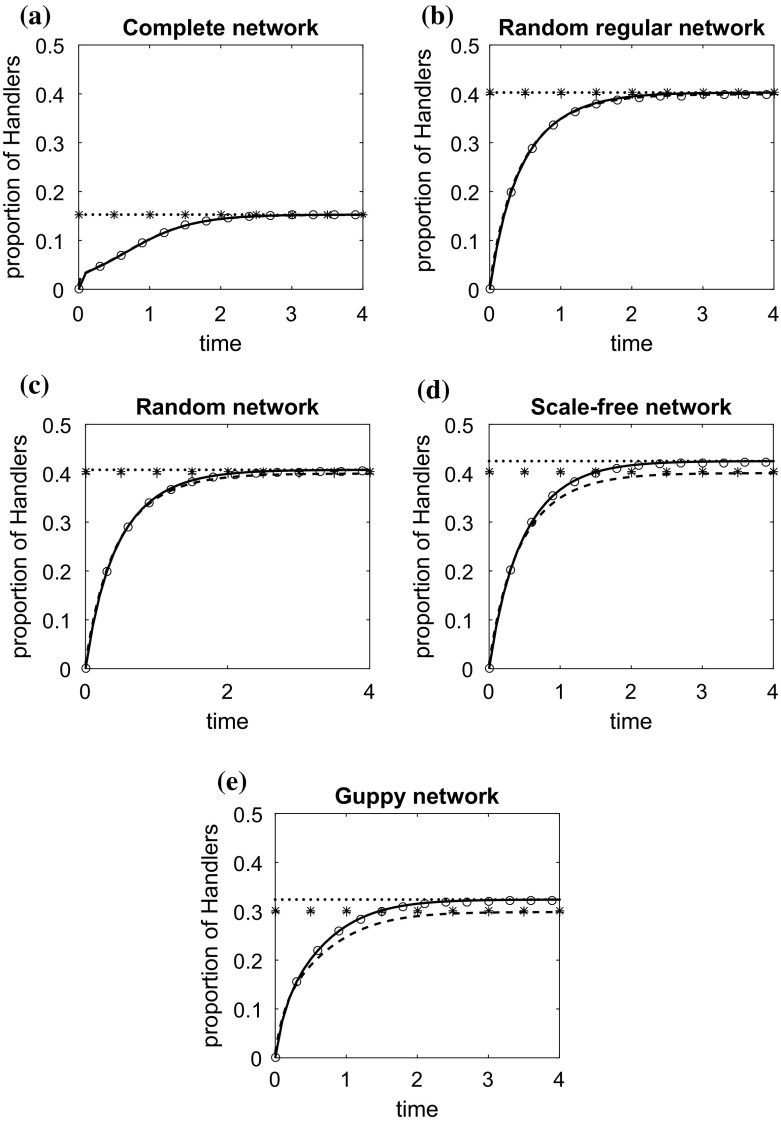
Change over time in the density of handlers on **a** a complete network, **b** a random regular network, **c** a random network, **d** a scale-free network, **e** the Guppy network. The characteristics of these networks are discussed in Sect. 4. The circles represent the average of 10,000 stochastic simulations. Initially, at $\hbox {time}=0$, all animals are at the searching state. The solid line is the solution of the individual-based model (9)–(11) and the dashed line the solution of the pairwise model (24)–(39). The dotted line and the asterisks, ‘*’, are respectively the approximate solutions (19) and (23) of the individual-based model at the steady state. The plus signs, ‘$+$’, is the solution of the pairwise model explicitly given in “Appendix A”. $P=1, t_{a,ij}/2=0.5, \alpha _{ij}=0.5, t_{h,i}=1, v_ff_i=1, v_{h,i}=0.3, i,j \in \{1,\ldots , N\}$

An example of the case where $v_{h,i}=v_h$, $\forall i\in \{1,\ldots , N\}$, is illustrated in Fig. 4. This example can describe a case in which foraging animals can search for animals handling a food item from their position by scanning the entire space, for example by rotating at rate 360 degrees per $v_h^{-1}$ seconds, and thus in the same amount of time they can see an arbitrary number of animals. However, their location or geographical position might affect the likelihood that they observe others. The animals can interact only if they can see each other, and a higher degree of a node in this case means a higher number of visible animals.

The structure effect is clearer here. The higher the degree of the network, the lower the density of handlers (see also Fig. 2b). As before, it is observed that in highly heterogeneous structures, the numerical and approximate analytical solutions of the individual-based model perform much better than those of the pairwise model, when compared with the results of stochastic simulations. However, on regular networks, or not very structurally heterogeneous populations, the pairwise model performs very well (and in some cases better than the individual-based model), demonstrating that it is a good compromise between accuracy and complexity.

### Case 2: inter-individual variability in parameter values

**Fig. 5:**
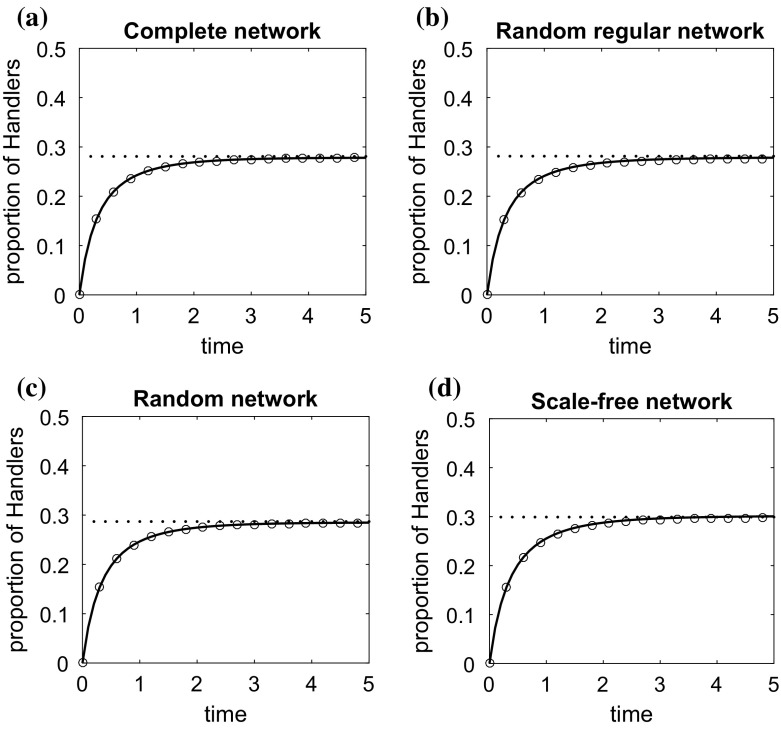
Change over time in the density of handlers on **a** a complete network, **b** a random regular network, **c** a random network, **d** a scale-free network. The characteristics of these networks are discussed in Sect. 4. The solid line is the solution of the individual-based model (9)–(11) and the circles represent the average of 10,000 stochastic simulations. Initially, at $\hbox {time}=0$, all animals are at the searching state. The dotted line is the approximate solution (19) of the individual-based model at the steady state. $t_{a,ij}^{-1}$, $t_{h,i}^{-1}$, $v_ff_i$, $i,j \in \{1,\ldots , N\}$, are drawn independently from an exponential distribution with mean equal to 1. $\alpha _{ij}$ is drawn from the uniform distribution on the unit interval [0, 1]. $v_{h,i}=1/d_i$

**Fig. 6:**
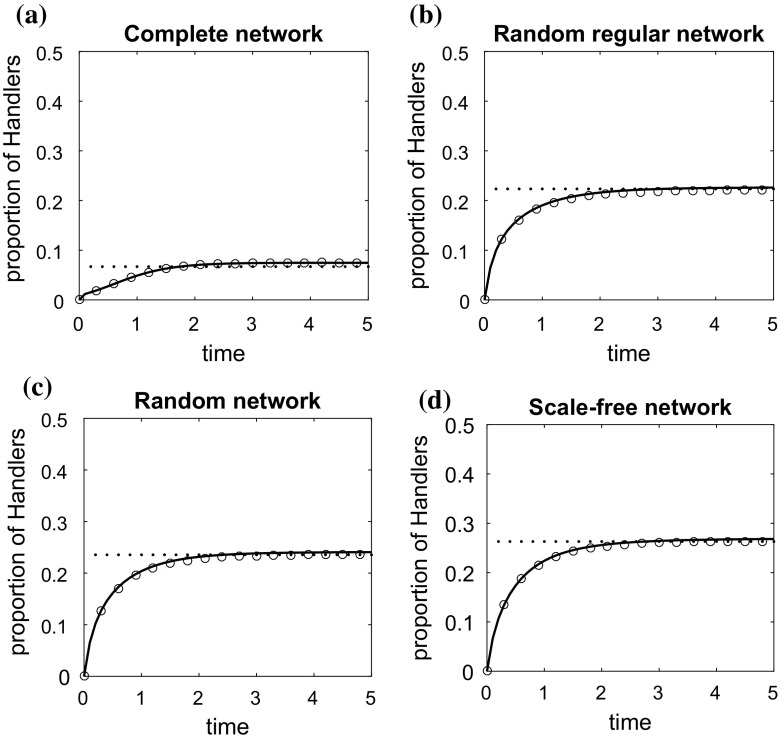
Change over time in the density of handlers on **a** a complete network, **b** a random regular network, **c** a random network, **d** a scale-free network. The characteristics of these networks are discussed in Sect. 4. The solid line is the solution of the individual-based model (9)–(11) and the circles represent the average of 10,000 stochastic simulations. Initially, at time$=$0, all animals are at the searching state. The dotted line is the approximate solution (19) of the individual-based model at the steady state. $t_{a,ij}^{-1}$, $t_{h,i}^{-1}$, $v_ff_i$, $v_{h,i}$, $i,j \in \{1,\ldots , N\}$, are drawn independently from an exponential distribution with mean equal to 1. $\alpha _{ij}$ is drawn from the uniform distribution on the unit interval [0, 1]

In order to illustrate the effectiveness of the individual-based model in highly heterogeneous environments, we also consider two different examples where the individual model parameters are not the same for every individual. In both examples, the individual parameters $v_ff_i$, $t_{h,i}^{-1}$, $t_{a,ij}^{-1}$, $i,j \in \{1,\ldots , N\}$, are drawn independently from an exponential distribution with mean equal to 1. $\alpha _{ij}$ is drawn from the uniform distribution on the unit interval [0, 1]. In the first example, illustrated in Fig. 5, $v_{h,i}=1/d_i$. In the second example, illustrated in Fig. 6, $v_{h,i}$ is also drawn from an exponential distribution with mean equal to 1. The parameter values for each individual are the same on the different structured populations, and we compare only the results on the complete network, the random regular network, the random network and the scale-free network that have the same population size.

In these examples, it makes sense to consider only the effectiveness of the individual-based model (9)–(11) developed in Sect. 3.1 when compared to the output of stochastic simulations. In both Figs. 5 and 6, it is observed that even in highly heterogeneous populations the individual-based model and its approximate solution (19) at the steady state predicts accurately the results of simulations in all networks considered.

## Discussion

We have extended the original model of kleptoparasitism proposed by Broom and Ruxton (1998) onto networks to study the effect of the population structure and other heterogeneities on the behaviour of kleptoparasitic populations. To describe the dynamics of a population placed on a network, we first developed an individual-based model which enabled us to incorporate individual-level mechanisms in the original model, for example to introduce heterogeneity in the consumption rates of animals, their ability to find food and other animals handling food, their fighting abilities, as well as in the fighting times over food between pairs of animals. Despite the accurate prediction of this model in describing the output of stochastic simulations, it consists of a large number of equations, which is $O({ dN})$. Assuming identical behavioural parameters and regular population structures, we also developed a pairwise model that has a significantly lower number of equations. For each model, we derived either approximate or exact solutions of the handling ratio, which is the rate at which an animal consumes food at the steady state. In highly heterogeneous structures the individual-based model performed much better than the pairwise model. However, in populations placed on regular or random networks, the solution of the pairwise model agreed very well with the results of stochastic simulations.

We have shown that the population structure may not greatly affect the dynamics of the population, mainly due to the fact that animals can discover and consume items of food independently of the population structure. The only effect of the structure is due to the change of the rate at which each animal is involved in aggressive interactions. Hence, in populations placed on a regular network, the decrease of the network degree has the same effect as that of the decrease of the rate at which foraging animals encounter handlers in a homogeneous well-mixed population. A more important influence of the population structure was observed on degree-heterogeneous structures, where the chance of encountering an animal and being engaged in a fight, either as a searcher or a handler, is not the same for every animal in the population, due to the different degree of connectivity. In particular, a significant effect was observed in scale-free networks, where the variance in the degree distribution is high. Clearly, in such networks, highly connected animals are more likely to fight over food with a neighbouring animal, affecting negatively their food consumption rate, whereas poorly connected animals handling a food item have a higher chance of consuming the food before being challenged by other animals. As the highly connected animals, and therefore the number of aggressive interactions among animals, on such structures are few, the average intake rate of the population increases compared to the respective rate in a well-mixed population.

This study is the first step in developing tools for the modelling of food-stealing interactions in more realistic settings, building upon models developed in idealised ‘well-mixed’ populations, to create models that can accommodate heterogeneous spatial and social relationships. In this first development of classic models of kleptoparasitism on networks animals do not have the option to optimise their foraging strategy and maximise their food intake rate in different ecological conditions. The influence of the population structure on the evolution of kleptoparasitic populations when animals can choose from a range of foraging strategies (see for example Broom et al. 2004) constitutes an interesting subject for subsequent studies. The general methodology could also apply more widely to related scenarios of animal interaction, such as that of predator interference (e.g. van der Meer and Ens 1997; Vahl 2006; van der Meer and Smallegange 2009).

## Appendix A: Steady state of the pairwise model

We assume that the system of differential Eqs. (24)–(38) is in a steady state, i.e. that all of the derivatives are set equal to zero. Firstly, we shall make some assumptions of independence within our system. We use these assumptions to find a solution, which we then verify is a solution of the original set of equations without the assumptions.

Given the relationship (42), we assume that

45 $$\begin{aligned}{}[{ HH}]=t_h\nu _{f}f[{ SH}], \end{aligned}$$

and that

46 $$\begin{aligned}{}[{ SS}]=\frac{1}{t_h\nu _{f}f}[{ SH}]. \end{aligned}$$

Clearly, the number of all pairs is equal to the number of links in the network, i.e.

47 $$\begin{aligned}&[{ SS}]+[{ HH}]+[{ AA}]+[{ RR}]+2\left( [{ AR}]+[{ AR}_J]+[{ SH}]\right. \nonumber \\&\qquad \left. +[{ SA}]+[{ SR}]+[{ HA}]+[{ HR}]\right) ={ dN}. \end{aligned}$$

Hence, all the singles and the pairs $[{ AR}_J]$, $[{ HH}]$ and $[{ SS}]$ can be expressed in terms of $[{ SH}]$, and given the expression (47) the system of equations with (24)–(38) set to equal 0 is reduced by eight equations.

Since $[A]=[R]$, at the steady state of the pairwise model, the equivalent independence assumptions to the above give $[{ SA}]=[{ SR}]$ and $[{ HA}]=[{ HR}]$.

Let us denote by [*F*], the number of animals involved in a fight, either as attackers *A* or defenders *R*. Hence, $[A]=[R]=[F]/2$, $[{ SA}]=[{ SR}]=[{ SF}]/2$, $[{ HA}]=[{ HR}]=[{ HF}]/2$, $[{ AR}_J]=[{ FF}_J]/2$ and $[{ AA}]+[{ RR}]+2[{ AR}]=[{ FF}]$, where $[{ AA}]=[{ RR}]=[{ AR}]=[{ FF}]/4$. From equation (28) and the moment closure approximation (39) we get that

48 $$\begin{aligned} \frac{2}{t_h}[{ SH}]+\frac{2}{t_a}[{ SF}]-2\nu _{f}f[{ SS}]-2\nu _{h}\left( \frac{d-1}{d}\right) \frac{[{ SS}][{ SH}]}{[S]}=0. \end{aligned}$$

Substituting Eqs. (44) and (46) in (48) we get

49 $$\begin{aligned}{}[{ SF}]=\left( \frac{d-1}{d}\right) \frac{\nu _{h}t_a\left( 1+t_h\nu _{f}f\right) [{ SH}]^2}{t_h\nu _{f}f\left( N-t_a\nu _{h}[{ SH}]\right) }. \end{aligned}$$

Similarly, from Eq. (29) and using (39) we get

50 $$\begin{aligned} 2\nu _{f}f[{ SH}]+\frac{2}{t_a}[{ HF}]-\frac{2}{t_h}[{ HH}]-2\nu _{h}\left( \frac{d-1}{d}\right) \frac{[{ HH}][{ HS}]}{[H]}=0. \end{aligned}$$

Substituting Eqs. (42), (44) and (45) in (50) we get

51 $$\begin{aligned}{}[{ HF}]=\left( \frac{d-1}{d}\right) \frac{\nu _{h}t_a\left( 1+t_h\nu _{f}f\right) [{ SH}]^2}{N-t_a\nu _{h}[{ SH}]}=0. \end{aligned}$$

From Eqs. (30)–(32) and the fact that $[{ FF}]=[{ AA}]+[{ RR}]+2[{ AR}]$ we get that at the steady state

52 $$\begin{aligned} 2\nu _{h}\left( [{ FSH}]+[{ FHS}]\right) -\frac{4}{t_a}[{ FF}]=0. \end{aligned}$$

Using the closure approximation (39) we get

53 $$\begin{aligned}{}[{ FF}]= \frac{t_a}{2}\nu _{h}\left( \frac{d-1}{d}\right) [{ SH}]\left( \frac{[{ SF}]}{[S]}+\frac{[{ HF}]}{[H]}\right) . \end{aligned}$$

Substituting (42), (44), (49) and (51) in (53) we get

54 $$\begin{aligned}{}[{ FF}]=\left( \frac{d-1}{d}\right) ^2\frac{\nu _{h}^2 t_a^2\left( 1+t_h\nu _{f}f\right) ^2[{ SH}]^3}{t_h\nu _{f}f\left( N-t_a\nu _{h}[{ SH}]\right) ^2}=0. \end{aligned}$$

Equation (34) can be written as

55 $$\begin{aligned} \frac{d[{ SH}]}{{ dt}}&= \nu _{f}f[{ SS}]+\frac{1}{t_h}[{ HH}]+\frac{1}{t_a}\left( [{ SF}]+[{ HF}]+[{ FF}_J]\right) \nonumber \\&\quad -\nu _{f}f[{ SH}]-\frac{1}{t_h}[{ SH}]-\nu _{h}\left( [{ SH}]+[{ SHS}]+[{ HSH}]\right) . \end{aligned}$$

Using the closure approximation (39) and substituting Eqs. (42), (44), (45), (46), (49), (51), $[{ FF}_J]=2[{ AR}_J]=2[A]$, where [*A*] is given by (41), and solving for $[{ SH}]$ we get that at the steady state

56 $$\begin{aligned}{}[{ SH}]=t_h\nu _{f}f q, \end{aligned}$$

where

57 $$\begin{aligned} q=\frac{{ dN}\left( Y \pm \sqrt{Y^2-4t_ht_a\nu _{f}f\nu _{h}Z}\right) }{2t_ht_a\nu _{f}f\nu _{h}Z }, \end{aligned}$$

and

58 $$\begin{aligned} Y&=t_h\nu _{f}f\left( 2dt_a\nu _{h}+ t_h\nu _{f}f+2 \right) + 1, \end{aligned}$$

59 $$\begin{aligned} Z&=t_h\nu _{f}f\left( d^2t_a\nu _{h}+ t_h\nu _{f}f+2\right) +1. \end{aligned}$$

Substituting (56) into Eqs. (40), (41), (42), (44), (45), (46), (49), (51) and (54) we get that the number of singles is given by

60 $$\begin{aligned}{}[S]=m,\qquad [H]=t_h\nu _{f}fm,\qquad [A]=[R]=\frac{[F]}{2}=\frac{t_ht_a\nu _{f}f\nu _{h}q}{2}, \end{aligned}$$

and the number of pairs by

61 $$\begin{aligned}{}[{ SS}]&=q, \end{aligned}$$

62 $$\begin{aligned}&=(t_h\nu _{f}f)^2 q, \end{aligned}$$

63 $$\begin{aligned}&=[{ RR}]=[{ AR}]=\frac{[{ FF}]}{4}=\left( \frac{d-1}{d}\right) ^2\frac{t_h^2t_a^2(\nu _{f}f)^2\nu _{h}^2q^3}{4m^2}, \end{aligned}$$

64 $$\begin{aligned}&=\frac{[{ FF}_J]}{2}=\frac{t_ht_a\nu _{f}f\nu _{h}q}{2}, \end{aligned}$$

65 $$\begin{aligned}&=[{ SR}]=\frac{[{ SF}]}{2}=\left( \frac{d-1}{d}\right) \frac{t_ht_a\nu _{f}f\nu _{h}q^2}{2m}, \end{aligned}$$

66 $$\begin{aligned}&=[{ HR}]=\frac{[{ HF}]}{2}=\left( \frac{d-1}{d}\right) \frac{t_h^2t_a(\nu _{f}f)^2\nu _{h}q^2}{2m}, \end{aligned}$$

where

67 $$\begin{aligned} m=\frac{N-t_ht_a\nu _{f}f\nu _{h}q}{1+t_h\nu _{f}f}. \end{aligned}$$

Substituting (57) into (67) we get

68 $$\begin{aligned} m=\frac{N(2Z-{ dY})\pm \sqrt{Y^2-4t_ht_a\nu _{f}f\nu _{h}Z}}{2Z\left( 1+t_h\nu _{f}f\right) }, \end{aligned}$$

where $2Z-{ dY}=(2-d)\left( t_h\nu _{f}f(t_h\nu _{f}f+2)+1\right)$. Hence, for $d>2$, ${ dY}>2Z$ and therefore, only the addition of the square root in (68) (the subtraction in expression (57)) can give a biologically plausible steady state.

We thus have a solution to the system based upon the assumptions from Eqs. (45) and (46), together with $[{ SA}]=[{ SR}]$ and $[{ HA}]=[{ HR}]$. Substituting into the original Eqs. (24)–(39) we find that this is a solution to this system. But is the solution unique? We have not been able to prove uniqueness. However, for reasons explained in Sect. 3.1.1, we believe that this is the unique solution of the pairwise model. Numerical investigation verifies that the system of differential Eqs. (24)–(38), together with the closure approximation (39), indeed converges to the above solution in all the cases that we have considered, something that is also supported by the output of stochastic simulations.

## Appendix B: The simulation model

Initially, all animals are at the searching state. In a small time interval $\delta t=0.001$, an animal in the searching state discovers a food item, independently of the network structure, with probability $1-\exp (-\nu _{f}f \delta t)$ (i.e. the number of food-discoveries in the time interval $\delta t$ is assumed to follow a Poisson distribution with associated parameter $\nu _{f}f \delta t$). Similarly, in the interval $\delta t$, a handler animal consumes a food item with probability $1-\exp \left( -t_h^{-1} \delta t\right)$. The probability of a searcher becoming engaged in a fight depends on the number of neighbouring animals handling a food item, $k_H$. This is taken to be equal to $1-\exp (- k_H \nu _{h} \delta t)$. Similarly, the probability of a handler being discovered by a searcher and engaged in a fight is equal to $1-\exp (- k_S \nu _{h} \delta t)$, where $k_S$ is the number of the neighbouring animals of the handler that are searching for food. Equivalently, a searcher is engaged in a fight with every neighbouring handler with probability $1-\exp (- \nu _{h} \delta t)$. A fight ends with probability $1-\exp (- (2/t_a) \delta t)$, and each of the animals obtains the food with probability $\alpha$.
